# Prevalence and Associated Factors of Tuberculosis in Prisons Settings of East Gojjam Zone, Northwest Ethiopia

**DOI:** 10.1155/2017/3826980

**Published:** 2017-10-17

**Authors:** Mucheye Gizachew Beza, Emirie Hunegnaw, Moges Tiruneh

**Affiliations:** ^1^Department of Medical Microbiology, School of Biomedical and Laboratory Sciences, College of Medicine and Health Sciences, University of Gondar, P.O. Box 196, Gondar, Ethiopia; ^2^Laboratory Section, Motta Hospital, Motta, Ethiopia

## Abstract

**Background:**

Tuberculosis, mainly in prisoners, is a major public health problem in Ethiopia where there is no medical screening during prison admission. This creates scarcity of TB data in such settings.

**Objective:**

To determine prevalence and associated factors of TB in prisons in East Gojjam Zone, Northwest Ethiopia.

**Methods:**

A cross-sectional study was conducted from February to May 2016 among 265 prisoners in three prison sites. Sputum was processed using GeneXpert MTB/RIF. Data were analyzed using SPSS version 20.0. Multivariable logistic regression was used; *p* values = 0.05 were considered statistically significant.

**Results:**

Of 265 prisoners, 9 (3.4%) were TB positive (males); 77.8%, 55.6%, and 55.6% of cases were rural dwellers, married, and farmers, respectively. Seven (2.6%) prisoners were HIV positive, and 3 (1.13%) had TB/HIV coinfection. One (0.4%) TB case was rifampicin resistant. Marriage (AOR = 1.5; 95% CI: 1.7, 13.03), HIV (AOR = 0.14; 95% CI: 0.001, 0.17), and sharing of rooms (AOR = 1.62; 95% CI: 2.6, 10.20) were predictors for TB.

**Conclusion:**

Nine prisoners were TB positive. One case showed rifampicin resistance and three had TB/HIV coinfection. Marriage, HIV, and sharing of rooms were predictors for TB. Prevention/control and monitoring are mandatory in such settings.

## 1. Background

Tuberculosis is caused by* Mycobacterium tuberculosis *(Mtb) and occasionally by an Mtb complex [1]. It is very common and is spread from person to person via air by droplet nuclei produced when a person with TB coughs, sneezes, talks, or sings [2]. One-third of the world's population are infected with Mtb; Eastern Europe, Southeast Asia, and sub-Saharan Africa exhibit the highest incidence [3]. In developing countries, 7% of all deaths are attributed to TB [4].

Tuberculosis has been recognized as a major public health problem in Ethiopia since the 1950s, and efforts to control it have continued since then [5], although it is an ancient disease that has affected mankind for more than 4,000 years [4]. Ethiopia ranks 7th in the world for TB burden and 3rd in Africa in 2008, with an estimated incidence of 378 new cases per 100,000 persons, 163 new smear positives per 100,000 persons, and a prevalence of 579 per 100,000 population [1]. It was the 3rd leading cause of patients' death (10.1%) in 2001 [6].

Tuberculosis in prisons usually exceeds 3,000 per 100,000 persons. Incidence and mortality rates owing to TB in prisons are higher [7]. Tuberculosis and HIV are two of the greatest health threats in African prisons [8]. The fast growing epidemic of TB in the prisoners in sub-Saharan Africa constitutes a threat to both inmates and the community [9]. There is an increasing recognition that the high risk of TB disease in prison settings poses a problem for prisoners and communities. Transmission in prisons is also particularly dangerous as it often involves resistant strains [10].

Studies carried out in Pakistan showed 48% latent TB cases among 425 prisoners [11] and 2.2% TB cases among 365 randomly selected imprisoned men [10]. Another study carried out in Khuzestan, Iran, revealed 7.9% TB cases among 4562 prison inmates [12] and 4.5% TB cases among 59 prisoners in Tajikistan [13]. Of 15,495 TB suspected inmates screened in 13 Ethiopian prisons, 765 (4.9%) had TB [14]. A study carried out in Eastern Ethiopian prisons revealed 8.9% TB cases among 371 prisoners [15], 19.4% in Gamo Gofa Zone [16], 1.83% in Hadiya zone [17], and 21.9% among 196 prisoners at the Bedele Woreda prison [18]. Another study conducted in prisons found in North Ethiopia also revealed that TB accounted for 8.0% in eight prisons [19], 10.4% from 250 prisoners [20], and 8.9% smear positive PTB [21].

A study done in Ghana revealed that HIV seroprevalence was 4.9% among prisoners [22]. A report in Abuja, Nigeria, prison depicted that HIV positivity was 6% among 200 prisoners [23]. A study conducted in a prison in Uganda showed that the prevalence of HIV was 4.1% among 248 prisoners [24]. Studies conducted in North Gondar prisons revealed that HIV positivity rates were 7.6% among 250 prisoners [20] and 12.3% among 114 TB patients [25].

Multidrug-resistant- (MDR-) TB cases in prisons are often higher than has been reported in the general population [7]. A report in Russia showed that the prevalence of isoniazid, rifampicin, streptomycin, ethambutol, and pyrazinamide resistance in new TB cases in prisoners was 38.0%, 25.2%, 34.6%, 14.7%, and 7.2%, respectively [26]. Another study done in Russian prison inmates indicated that rifampicin resistance was 58.2% among 140 prisoners [27]. MDR-TB also remains a critical health problem in Ethiopia and its prevalence was 1.6% and 11.8% among new and retreatment cases, respectively [28]. Another report in Amhara National Regional State, Ethiopia, also indicated that rifampicin resistance rate was 2.8% among 606 suspects of MDR-TB (Nigus et al., 2015).

People in congregated settings like prisons and refugee camps are at high risk for acquisition of TB due to overcrowding, close living conditions, insufficient ventilation, low socioeconomic status, and poor nutrition and health of prison inmates [10, 29, 30]. A Pakistani report revealed that prisoners' educational level, smoking status, and duration of current incarceration in prison were found to be (*p* < 0.05) predictors of Mtb infection [11]. A study indicated that most prison settings encounter a variety of challenges which hinder TB control: insufficient laboratory capacity and diagnostic tools, interrupted supply of medicines, weak integration between civilian and prison TB services, inadequate infection control measures, and low policy priority for prison healthcare [11].

Various studies in Ethiopian prison settings also explained that educational status, HIV, diabetes, malnutrition, alcoholism, smoking cigarettes, poverty, and being in prison, history of TB treatment, TB contact history, cough > 2 weeks before diagnosis, night sweating, decreased body mass index, poor ventilation of the room, and length of imprisonment were the commonest identified risk factors for TB [16, 18–20, 31].

## 2. Materials and Methods

### 2.1. Study Area

This study was carried out in East Gojjam Zone, in three prison sites: Motta, Bichena, and Debre Markos. The prisons serve as a central destination for inmates coming from many surrounding smaller prisons or police stations. Selection of these prisons for the study was based on their role as central prisons, easy for sample transportation and cooperation from the relevant administrative body. Based on the recent year's national census, East Gojjam Zone has a total population of 1,670,685, of whom 827,145 are men. The total prisoners in this zone were 2700, of whom 2628 were men. This zone has one referral hospital, three currently working primary hospitals, 101 health centers, 406 health posts, 1 higher clinic (private), 21 medium private clinics, and 3 specialty clinics.

### 2.2. Study Design and Period

An institution based cross-sectional study was conducted from February to May 2016.

### 2.3. Population

#### 2.3.1. Source Population

The source population included all prisoners in East Gojjam Zone at the time of the study.

#### 2.3.2. Study Population

The study population included all prisoners who had cough for more than two weeks during the study period.

### 2.4. Inclusion and Exclusion Criteria

#### 2.4.1. Inclusion Criteria

Study participants who had cough for more than two weeks were included.

#### 2.4.2. Exclusion Criteria

Those who failed to give sputum specimens and consent were excluded.

### 2.5. Variables

#### 2.5.1. Dependent Variables

Prevalence of tuberculosis was the dependent variable.

#### 2.5.2. Independent Variables

Independent variables were age, sex, marital status, duration of cough, place of residence before incarceration, occupation before imprisonment, educational status, number of prisoners per cell, length of stay in the prison, previous anti-TB treatment, diabetes mellitus, HIV status, cigarette smoking, and sharing of cells with TB patients.

### 2.6. Sample Size Determination and Sampling Technique

#### 2.6.1. Sample Size

Sample size was determined using a single population proportion formula by assuming a prevalence of 19.4% [16].

(1) n=Zα/22P1−Pd2,n=1.9620.1941−0.1940.052,n=241,

where *n* is the sample size, *Z* is the *z*-score for 95% CI (1.96), *P* is the prevalence (19.4%), and *d* is the margin of error (5%).

Adding a 10% nonresponse rate gave us 265. The number of study participants from each study area was calculated as follows: number of prisoners in Debre Markos (*n*1) = *nf*/*N* × *n*1 = 265/2700 × 1300 = 128; number of prisoners in Bichena (*n*2) = *nf*/*N* × *n*2 = 265/2700 × 880 = 86; and number of prisoners in Motta (*n*3) = *nf*/*N* × *n*3 = 265/2700 × 520 = 51, where *nf* is the final sample size, *ni* is the size of that prison, and *N* represents total prisoners.

#### 2.6.2. Sampling Technique

There were 2700 prisoners in the specified prison sites during the study period. Health committee members present in each cell were given assignments of recruiting prisoners for the study. They were oriented to recruit inmates who had cough > 2 weeks, and health professionals interviewed them in prison clinics. Each study participant was selected by a convenient sampling technique.

### 2.7. Data Collection and Laboratory Investigation

Semistructured and pretested questionnaires were used to collect sociodemographic characteristics and associated factors. Questionnaires were prepared in English and then translated to Amharic and then back to English. Prior to the beginning of data collection, all data collectors were trained on an overview of the assessment and the objectives of the study.

About 1–4 ml of a purulent (mucoid) sputum specimen was collected from prisoners using a sterile falcon tube according to the recommended guideline. Specimens were stored at a maximum temperature of 35°C for less than three days and in a 4°C refrigerator for 4–10 days until transported to GeneXpert MTB/RIF diagnosis site. The stored sputum sample of each inmate was put in one triple pack container and transported in an icebox within five days of the sample collection to the GeneXpert MTB/RIF laboratory site and analyzed as soon as possible by using the recommended GeneXpert MTB/RIF test protocol [28, 32, 33]. HIV rapid testing was done as per the national HIV testing algorithm guideline [34].

### 2.8. Quality Control

Pretest was done on individuals who have similar characteristics to this study participant at Bahir Dar Prison to standardize the questionnaire. Completeness of data was cross-checked to ensure whether the right data were entered correctly. Laboratory procedures were performed by following SOPs for GeneXpert MTB/RIF assay. Sample processing control (SPC), probe check control, and internal quality control (IQC) have been always done by the machine itself. In addition, HIV test quality was checked by using previously known HIV positive and negative samples. All materials, equipment, and procedures were adequately maintained and calibrated.

### 2.9. Data Processing and Analysis

Collected data were computerized using Excel, cleaned, and entered to be analyzed using SPSS version 20.0. Logistic regression was used to explain the effect of independent variables on the outcome variable. *p* values ≤ 0.05 were considered statistically significant.

### 2.10. Definitions of Terms

#### 2.10.1. Pulmonary Tuberculosis

Pulmonary tuberculosis is a disease caused by* M. tuberculosis* that mainly affects the lungs.

#### 2.10.2. Rifampicin Resistance

Rifampicin resistance is a surrogate marker of MDR-TB (rifampicin monoresistance tuberculosis).

#### 2.10.3. Associated Risk Factors

These include any attribute, characteristic, or exposure of an individual that increases the likelihood of developing TB.

#### 2.10.4. MDR-TB

This is a form of TB infection caused by bacteria that are resistant to treatment with at least two of the most powerful first-line anti-TB medications (drugs): isoniazid and rifampin.

### 2.11. Ethical Considerations

The study was started after ethical clearance was obtained from the Ethical and Research Committee of the School of Biomedical and Laboratory Sciences. Written consent was obtained from the study participants. Each confirmed TB case was linked to the health institutions for anti-TB treatment. Pre- and post-HIV test counseling was provided by nurses after written consent and HIV positives were linked to ART clinic. Confidentiality of study participants' data was kept by using code number and locking it in a cupboard and on a computer locked with a password.

## 3. Results

### 3.1. Sociodemographic Characteristics of the Study Participants

A total of 265 prisoners were recruited in this study, of whom 263 were males. Mean age of the prisoners was 36.5 years with the range of 15 to 72 years, having a normal distribution. Majority (83%) of the prisoners stayed in the prison for one year; 215 (81.5%) were imprisoned from rural areas; 261 (98.5%) were living in prison cells whose windows have been closed. Nearly half of the prisoners (49.1%) were attending primary school; 20 (83%) were married; 208 (78.5%) were farmers, and 258 (97.4%) were living under the range of 1–89 individuals per cell (Table 1).

### 3.2. Prevalence of Tuberculosis among Prisoners

Among 265 prisoners, 9 (3.4%) were positive for Mtb by GeneXpert MTB/RIF, and all were males. Of these, 7 (77.8%) were detected to be rural dwellers. Majority (55.6%) of the TB cases were married. All TB cases were from nonventilated cells where windows were closed all the time and all cases had TB symptoms after admission to the prison (Table 2).

Seven (2.6%) prisoners were HIV positive and all were males. Of these, three cases had TB coinfections. Of all HIV cases, 4 (57.1%) were > 30 years of age. Four (57.1%), 6 (85.7%), and 4 (57.1%) HIV cases were illiterate, farmers, and urban dwellers, respectively (Table 2). About 0.4% of the prisoners had rifampicin resistant Mtb.

### 3.3. Factors Associated with Tuberculosis among Prisoners

Using binary and multivariable logistic regression analysis, we found that marital status (COR = 4.9, 95% CI = 1.258–19.194; AOR = 1.5, 95% CI = 1.7–13.03), HIV status (COR = 31.5, 95% CI = 5.74–172.74; AOR = 0.14, 95% CI = 0.001–0.17), and sharing of cells with TB patients (COR = 4.83, 95% CI = 1.14–20.56; AOR = 1.62, 95% CI = 2.6–10.20) in the prison were significantly associated with TB positivity (Table 2). This meant that the odds of having TB infections among the married prisoners were 1.5 [AOR = 1.5, 95% CI: 1.7–13.03] times higher than in those who were not married. Similarly, the odds of having TB infections among those study participants who shared living cells/rooms with TB patients were 1.6 [AOR = 1.62, 95% CI: 2.6–10.20] times more than in those who did not have such an experience. Lastly, being HIV negative among prisoners was 86% more likely to be protected from TB infections as compared to those prisoners who had HIV infection [AOR =** 0.14**, 95% CI:** 0.001–0.17**].

## 4. Discussion

This study was aimed at determining the prevalence and associated factors of TB infection in the prison settings in East Gojjam Zone, Northwest Ethiopia. Prevalence of TB in prisoners is usually higher than the national average [35]. However, our study indicated that the prevalence of TB among prisoners in East Gojjam Zone has been found to be lower than in the previous studies conducted in the Amhara National Regional State (8.0%) [19], in North Gondar Zone prisons (10.4% [20] and 8.9% [21]), and in Eastern Ethiopian Prison (8.9%) [15]. The possible reasons for the difference might be associated with the variation of the diagnostic methods used, such as sputum microscopy, culture, and GeneXpert by the previous study and GeneXpert in our case. This low prevalence may also mean that there might be a comparatively good TB infection control in the prison management system of East Gojjam prisons. The higher rates in other cases may also mean that there were no good TB control systems in such a setting. On the other hand, our prevalence rate is higher than in the studies conducted on prisoners in Ghana (0.9%) [36] and Uganda (2.0%) [24]. The probable explanation for this difference might be due to the variation of study settings, study population, diagnostic methods used, and better TB control systems in these countries than in Ethiopia.

The prevalence rate of the present study was nearly comparable to the results of the study done on prisons in Ethiopia (4.9%) [16], Tajikistan (4.5%) [13], and South Africa (3.5%) [37]. The possible explanation of the similarity might be that whatever different methodologies they may have used, they may have implemented good comparable preventive and diagnostic strategies in their prison settings.

Our study showed low TB prevalence when compared with the study from Gamo Gofa zone (19.4%) [16]. The possible explanation for this difference might be due to the diagnostic methods employed, which included sputum microscopy and culture in Gamo Gofa and GeneXpert in our case. The potential reason for this difference may mean also that there are no good TB control systems in the prisons of Gamo Gofa. Tuberculosis prevalence among prisoners in our study was lower than the prevalence in Nigeria (54.2%) [38], Pakistan (48%) [11], and Southern Iran (7.9%) [12]. High rates of TB in Nigeria and Pakistan were not comparable with that of ours: it seems that there are no systems of initial screening for TB and prisoners' health services in such prisons.

Prevalence of HIV infection in our study was lower than in the reports from prisons in Gondar (7.6%) [20], Ghana (4.9%) [22], Nigeria (6%) [23], Uganda (4.1%) [24], and North Gondar (12.3%) [25]. The difference is possibly due to the discrepancies in testing algorithms.

Rifampicin resistant prevalence in the current study was lower than the result of the previous study conducted in the Amhara National Regional State (2.8%) [39]. The finding of our study was much lower than the reports in Russia (25.2% [26] and 58.2% [27]). The possible explanation might be that in Russia they may have had very high rates of MDR-TB. Dramowski et al. [40] reported that rifampicin resistant TB is increasingly encountered particularly among HIV infected children in Cape Town province. Saunders et al. [41] reported in Bujumbura the appearance of MDR-TB and rifampicin resistance in new cases. Toraore et al. [42] reported rifampicin resistance in Mtb isolates from diverse countries by a commercial line probe assay as an initial indicator of MDR-TB. Hence, rifampicin resistance is still infrequent in our region as a proxy of MDR-TB.

Various factors make prisoners in the prison settings more susceptible to TB infection. Our results revealed that HIV positivity, sharing of cells with TB patients, and marital status have a statistically significant association with prevalence of TB (Table 1).

## 5. Conclusion

Prevalence of TB in East Gojjam Zone prison inmates is 2.4 times less than the prevalence in the general population of Amhara National Region State (8%). The HIV prevalence (2.6%) in prison inmates of East Gojjam Zone is slightly higher as compared to the national HIV prevalence (1.14%) reported in 2014. The number of rifampicin resistant TB cases found in the current study was 1 (0.4%), which is less than that reported in the Amhara National Regional State (2.8%). Furthermore, the factors associated with TB positivity in the present study included HIV status, sharing of cells/living rooms with TB patients, and marital status. Results observed in East Gojjam Zone prisons confirm that TB disease exists among prisoners. These findings are serious problems that should no longer be ignored. Prisoners are the most vulnerable individuals, yet the most marginalized population in our society. Thus, the following recommendations are suggested: (1) screening of all new prisoners for TB and HIV before they join; (2) implementing appropriate interventions immediately to control the development and transmission of TB disease in this setting and prevent the spread of TB to the general population; (3) periodic TB screening especially with the use of GeneXpert MTB/RIF test which allows for the timely diagnosis as well as treatment initiation, which is critically important among inmates, so as to effectively control the spread of TB disease in prisons; and (4) precautions against the development of MDR-TB since rifampicin resistance is a probable indicator for the development of MDR-TB though the observation in our study seems to be small.

## Figures and Tables

**Table 1:** Sociodemographic characteristics of prison inmates in East Gojjam Zone (*n* = 265), 2016.

Variable	Response	Frequency	Percent (%)
Age in years	15–30 >30	98 167	37.0 63.0
Sex	Male Female	263 2	99.2 0.8
Marital status	Single Married Divorced	39 220 6	14.7 83.0 2.3
Educational status	Uneducated Primary school Secondary school Tertiary school	102 130 31 2	38.5 49.1 11.7 0.8
Residence	Rural Urban	215 50	81.1 18.9
Occupation	Farmer Government employed Self-employed Student	208 11 27 19	38.5 49.1 11.7 0.8
Length of stay in prison	<2 months 2–6 months 7–12 months >12 months	12 22 11 220	4.5 8.3 4.2 83.0
Number of prisoners per cell	1–89 >89	258 7	97.4 2.6

**Table 2:** Prevalence of TB among prisoners (*n* = 265) using GeneXpert MTB/RIF assay, with multivariable analysis showing its associated predictors in East Gojjam Zone, 2016.

Variable	Response	TB status		COR [95% CI]	AOR [95% CI]	*p* value
		*TB positive *(*n* = 9)	*TB negative *(*n* = 256)			
Age in years	≤30	5 (1.88%)	98 (36.9%)	2.13 [0.56–8.12]	0.53 [0.10–2.83]	0.460
	>30	4 (1.50%)	167 (63%)	1	1	
Marital status	Single Married	4 (1.50%) 5 (1.88%)	35 (13.5%) 221 (83.4%)	**1** **4.9 (1.258, 19.194) **	**1** **1.5 [1.7–13.03]**	**1** **0.01**^*∗*^
Educational status	Illiterate	2 (0.8%)	100 (37.7%)		7.63 [0.55–105.9]	0.139
	Primary, secondary, and above	4 (1.50%) 3 (1.23%)	126 (47.5%) 30 (11.3%)		3.54 [0.48–25.73]	0.212
					1	
Residence before incarceration	Rural	7 (2.64%)	208 (78.5%)	0.81 [0.16–4.01]	0.46 [0.47–4.6]	0.511
	Urban	2 (0.75%)	48 (18.1%)	1	1	
Length of stay in prison in months	≤12	1 (0.37%)	44 (16.6%)	0.60 [0.073–5.0]	0.9 [0.074–10.9]	0.933
	>12	8 (3.01%)	212 (80%)	1	1	
Number of prisoners per cell	1–89	9 (3.39%)	249 (93.9%)	—	—	0.615
	>89	0 (0%)	7 (26.4%)			
Ventilation status of the cells	Window closed at all times	9 (3.39%)	252 (95%)	—	—	0.706
	Window opened sometimes	0 (0%)	4 (1.5%)			
Sharing cells with TB patients	Yes No	3 (1.23%) 6 (2.26%)	24 (9.05%) 232 (87.5%)	**4.83 [1.14–20.56]** **1**	**1.62 [2.6–10.20]** **1**	**0.02** ^*∗*^ **1**
Duration of cough in weeks	2–4	1 (0.37%)	102 (38.4%)	0.19 [0.02–1.53]	8.31 [0.67–102.6]	0.099
	>4	8 (3.01%)	154 (58.1%)	1	1	
Time of occurrence of cough	Before imprisonment	0 (0%)	14 (5.2%)	—	—	0.741
	After imprisonment	9 (3.39%)	242 (93.4%)			
TB symptoms before admission to the prison	Yes	0 (0%)	19 (7.1%)	—	—	0.396
	No	9 (3.39%)	237 (89.4%)			
Cigarette smoking	Yes	0 (0%)	4 (1.5%)	—	—	0.706
	No	9 (3.39%)	252 (95%)			
Nutritional status	Normal	9 (3.39%)	253 (95.4%)	—	—	0.471
	Abnormal	0 (0%)	3 (1.23%)			
Diabetes mellitus	Yes	0 (0%)	4 (1.5%)	—	—	0.706
	No	9 (3.39%)	252 (95%)			
HIV status	Positive Negative	3 (1.23%) 6 (2.26%)	4 (1.5%) 252 (95%)	**31.5 [5.74–172.74]** **1**	**0.14 [0.001–0.17]** **1**	**0.001** ^*∗*^ **1**
Previously treated TB	Yes	0 (0%)	22 (8.3%)	—	—	0.358
	No	9 (3.39%)	234 (88.3%)			

*∗* indicates *p* value < 0.005.
