# Erratum to “Prevalence and Associated Factors of Tuberculosis in Prisons Settings of East Gojjam Zone, Northwest Ethiopia”

**DOI:** 10.1155/2018/1020349

**Published:** 2018-08-09

**Authors:** Emirie Hunegnaw, Moges Tiruneh, Mucheye Gizachew

**Affiliations:** ^1^Laboratory Section, Motta Hospital, Motta, Ethiopia; ^2^Department of Medical Microbiology, School of Biomedical and Laboratory Sciences, College of Medicine and Health Sciences, University of Gondar, P.O. Box 196, Gondar, Ethiopia

In the article titled “Prevalence and Associated Factors of Tuberculosis in Prisons Settings of East Gojjam Zone, Northwest Ethiopia” [1], the order of the authors was incorrect. Additionally, the name of the corresponding author was given incorrectly as Mucheye Gizachew Beza. The author's name should have been written as Mucheye Gizachew. The revised authors' list and affiliations are shown above.
